# From skin aging biomarkers to precision esthetic longevity: a translational diagnostic framework

**DOI:** 10.3389/fmed.2026.1902741

**Published:** 2026-08-14

**Authors:** Sarit Cohen, Dominik Thor

**Affiliations:** 1 Department of Plastic and Reconstructive Surgery, Shamir Medical Center, Zerifin, Israel; 2 Gray Faculty of Medical & Health Sciences, Tel Aviv University, Tel Aviv, Israel; 3 Geneva College of Longevity Science, Geneva, Switzerland; 4 Mayo Clinic, Centre for Aesthetic Medicine & Surgery, Rochester, MN, United States; 5 Department of Geriatrics and Gerontology, Carol Davila University of Medicine and Pharmacy, Bucharest, Romania

**Keywords:** biological age, biomarkers, digital phenotyping, epigenetic clocks, esthetic longevity, precision dermatology, skin aging, translational diagnostics

## Abstract

Skin aging is commonly evaluated through visible phenotype, clinical grading, and photographic documentation, yet these approaches incompletely capture the biological mechanisms that shape tissue quality, regenerative capacity, and long-term response to intervention. Recent advances in longevity science, including skin-specific epigenetic clocks, molecular profiling, functional tissue assessment, imaging technologies, and artificial intelligence-derived digital phenotyping create an opportunity to redefine esthetic dermatology as a more biologically informed and longitudinally guided discipline. This article combines a critical synthesis of the current literature with an original translational diagnostic framework for precision esthetic longevity, integrating five complementary layers: systemic biological age and longevity biomarkers; skin- and tissue-specific molecular biomarkers, including epigenetic, senescence-related, inflammatory, glycation, oxidative damage, extracellular matrix, and barrier-related domains; functional biomarkers reflecting elasticity, hydration, microcirculation, barrier integrity, and healing capacity; imaging-based and morphometric diagnostics capturing structural, optical, and spatial features of skin and facial aging; and digital phenotyping tools that may support standardized assessment and longitudinal monitoring. The central premise is that esthetic aging should not be interpreted as a single surface phenotype, but as the visible and measurable expression of interacting systemic, molecular, functional, structural, and digital dimensions. Biological age biomarkers and epigenetic clocks may provide systemic context, whereas skin-specific molecular and imaging biomarkers may offer more direct insight into local tissue aging. However, most available tools remain insufficiently validated for esthetic treatment selection or claims of biological rejuvenation. We therefore emphasize analytical validity, clinical validity, reproducibility, standardization, phototype-inclusive validation, and clinically meaningful endpoints as prerequisites for responsible translation. To make this actionable, we outline a methodological road map for prospectively validating the framework in future multilayer biomarker studies. A layered diagnostic model may support the transition from appearance-based assessment toward mechanism-informed esthetic medicine while maintaining caution regarding overinterpretation, commercialization, and premature clinical adoption.

## 1 Introduction

Skin aging is a visible, measurable, and biologically complex process shaped by the interaction of intrinsic aging, environmental exposure, genetic susceptibility, metabolic status, inflammation, oxidative stress, hormonal changes, and tissue-specific regenerative capacity. In dermatology and esthetic medicine, aging has traditionally been assessed through clinical examination, standardized photography, grading scales, and evaluation of visible features such as wrinkles, laxity, pigmentation, erythema, texture irregularity, volume loss, and perceived facial age. These approaches remain essential for clinical communication and treatment planning, but they primarily describe phenotype. They do not fully capture the molecular, functional, structural, and systemic processes that determine why tissues age differently, how they respond to intervention, and whether apparent improvement reflects transient visual change or more sustained biological remodeling (1–4).

The emergence of longevity science has shifted the understanding of aging from a chronological process to a biologically heterogeneous and potentially measurable trajectory (5). Biological age biomarkers, epigenetic clocks, inflammatory and metabolic signatures, cellular senescence markers, glycation-related parameters, oxidative damage indicators, and multi-omics approaches have created new opportunities to assess aging beyond chronological age (5–8). In parallel, dermatology has increasingly incorporated quantitative methods to evaluate skin aging, including molecular profiling, barrier assessment, elasticity measurement, microcirculatory testing, high-resolution imaging, three-dimensional morphometry, and digital image analysis. These developments suggest that skin aging and esthetic tissue decline may be interpreted through a broader diagnostic lens that integrates systemic aging biology with local cutaneous and structural tissue changes.

However, translating longevity biomarkers into esthetic dermatology presents several challenges. Most biological age clocks and systemic biomarkers were developed to predict morbidity, mortality, physiological decline, or healthspan-related outcomes rather than localized skin aging or esthetic treatment response. Conversely, many skin-analysis devices and digital platforms quantify visible features such as pigmentation, wrinkles, pores, redness, texture, or apparent age, but their outputs do not necessarily represent validated biological biomarkers (7, 8). A measurement may be reproducible and visually persuasive without being clinically valid, biologically specific, or useful for treatment selection. This distinction is particularly important in esthetic medicine, where commercial enthusiasm and patient demand may outpace evidence, and where claims of skin age, biological rejuvenation, or regenerative response require careful scientific interpretation (7, 8).

Precision dermatology requires biomarkers that are not only measurable, but analytically reliable, biologically meaningful, clinically interpretable, and useful within a defined context (7, 8). For esthetic longevity, this context includes assessment of tissue quality, regenerative capacity, inflammatory tendency, barrier integrity, extracellular matrix remodeling, vascular support, structural aging, procedural recovery, and durability of treatment response. No single biomarker is likely to capture this complexity. Similar visible phenotypes may arise from different biological mechanisms, while similar biological processes may manifest differently across patients depending on phototype, environmental exposure, anatomy, lifestyle, systemic health, and prior interventions. This inherent heterogeneity supports the need for a layered and integrative diagnostic model rather than a single biomarker panel or universal aging score.

**Figure 1:**
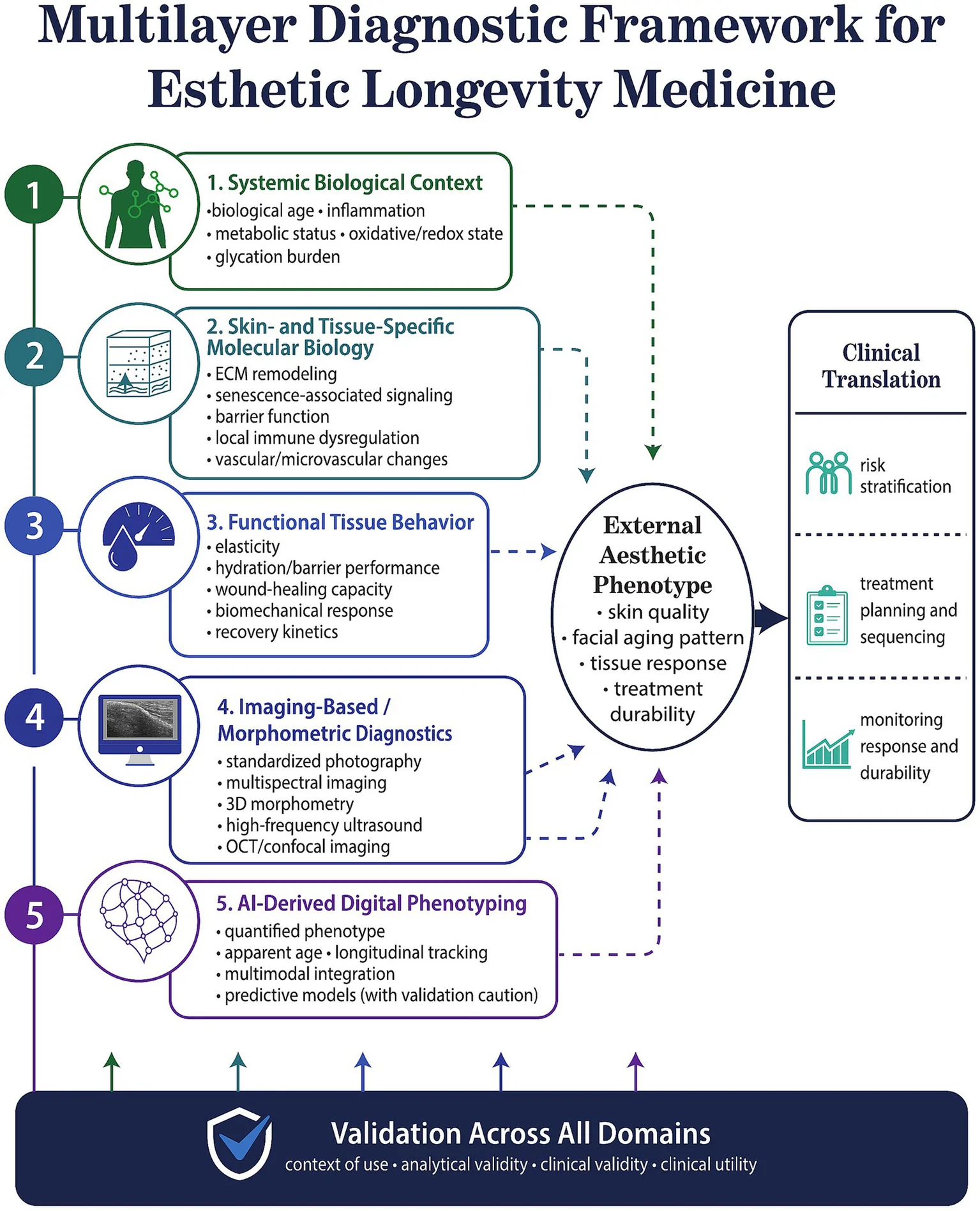
The proposed framework integrates five complementary diagnostic layers: systemic biological context, skin- and tissue-specific molecular biology, functional tissue behavior, imaging-based and morphometric diagnostics, and AI-derived digital phenotyping. These domains converge into the external esthetic phenotype, including skin quality, facial aging pattern, tissue response, and treatment durability. Across all layers, clinical translation requires defined context of use, analytical validity, clinical validity, and clinical utility. This layered model is intended to inform risk stratification, treatment planning, and longitudinal monitoring of response and durability once prospectively validated; it does not by itself establish how an individual biomarker should change management in a defined patient group, while emphasizing caution against overinterpretation of unvalidated biomarkers or premature claims of biological rejuvenation.

This article provides a critical synthesis of the emerging literature on biomarkers and diagnostic modalities relevant to precision esthetic longevity and proposes a translational diagnostic framework that integrates five complementary domains: systemic biological age and longevity biomarkers; skin- and tissue-specific molecular biomarkers, including epigenetic, senescence-related, inflammatory, glycation, oxidative damage, extracellular matrix, and barrier-related domains; functional tissue biomarkers; imaging-based and morphometric diagnostics; and artificial intelligence-derived digital phenotyping. Together, these domains provide a structured approach for linking external esthetic phenotype with systemic biological context, local cutaneous biology, tissue function, structural change, and longitudinal treatment response (Figure 1 and Table 1).

**Table 1:** Multilayer diagnostic framework for precision esthetic longevity medicine.

Diagnostic layer	Representative biomarkers/tools	Biological/clinical domain assessed	Potential clinical role	Key validation limitation
Systemic biological context	Epigenetic clocks (systemic and skin-specific), inflammatory markers, metabolic markers, oxidative/redox markers, glycation burden	Systemic aging burden, inflammatory-metabolic status, and physiological reserve	Context for healing capacity, risk stratification, systemic resilience	Mostly not validated as direct markers of facial or skin aging
Skin- and tissue-specific molecular biology	ECM markers, senescence markers, local cytokines, barrier markers, vascular/microvascular markers, genetic determinants	Local cutaneous, dermal, and connective-tissue aging biology	Mechanistic interpretation of tissue quality and remodeling potential	Often research-based; limited clinical thresholds and sampling standardization
Functional tissue behavior	TEWL, hydration, elasticity, microcirculation, wound-healing kinetics	Dynamic tissue performance, barrier competence, and recovery capacity	Pre-procedural optimization, recovery prediction, monitoring of tissue resilience	Protocol-dependent; limited longitudinal esthetic outcome validation
Imaging-based/morphometric diagnostics	Standardized photography, multispectral imaging, 3D morphometry, ultrasound, OCT, confocal imaging, multiphoton microscopy, LC-OCT	Structural, optical, spatial, and morphometric expression of aging	Documentation, treatment planning, longitudinal response assessment	Device-, operator-, and protocol-dependent; often indirect biological interpretation
AI-derived digital phenotyping	Quantified phenotype, apparent age, automated wrinkle/texture/pigment analysis, longitudinal tracking	Digitally quantified visible phenotype and pattern change	Standardization, monitoring, multimodal integration, possible future prediction	Requires external validation, bias assessment, transparency, and proof of clinical utility

3D, three-dimensional; AI, artificial intelligence; ECM, extracellular matrix; LC-OCT, line-field confocal optical coherence tomography; OCT, optical coherence tomography; TEWL, transepidermal water loss.

By reframing esthetic aging as the visible and measurable expression of interacting systemic, molecular, functional, structural, and digital dimensions, this review aims to bridge skin aging biology, longevity science, and precision dermatology. The proposed framework may support the transition from appearance-based assessment toward mechanism-informed esthetic medicine by improving biomarker selection, endpoint development, longitudinal monitoring, and translational study design (7–14). At the same time, it does not imply that currently available biomarkers provide definitive treatment algorithms, validated rejuvenation measures, or stand-alone clinical decision tools. Responsible translation requires analytical validity, clinical validity, reproducibility, phototype-inclusive validation, clinically meaningful endpoints, and caution regarding overinterpretation, premature commercialization, and unvalidated claims of biological rejuvenation (7–11).

## 2 Search strategy

This narrative review was conducted following Preferred Reporting Items for Systematic Reviews and Meta-Analyses (PRISMA) 2020 principles where applicable to a non-systematic review. A structured literature search was performed in PubMed/MEDLINE and Google Scholar for publications from 2015 to 2026; a small number of foundational references outside this window were retained where required for conceptual grounding. Search queries combined core concepts from skin-aging biology and esthetic medicine — biomarkers and diagnostics, cellular senescence, epigenetic clocks and DNA methylation, inflammaging, and esthetic/cosmetic dermatology.

The search identified 10,311 records (1,187 in PubMed/MEDLINE and 9,124 in Google Scholar). Because Google Scholar returns a high volume of low-specificity results — including duplicates, citations, and non-peer-reviewed items — records were screened in relevance-ranked order rather than exhaustively. After removal of duplicates and of records that were non-peer-reviewed, purely promotional or commercial, off-topic, or not retrievable in full text, titles and abstracts were screened for relevance to one or more of the five diagnostic domains, and shortlisted records were assessed in full text. Reference lists of key reviews and consensus documents were screened for additional eligible studies (backward citation searching).

Inclusion criteria were peer-reviewed, English-language publications relevant to one or more of the five diagnostic domains, comprising primary studies, methodological or consensus statements, and regulatory framework documents. Records were excluded if they were non-peer-reviewed, purely promotional or commercial, duplicative, or not retrievable in full text. Selection prioritized methodological quality and representativeness across domains over exhaustive coverage.

A final corpus of 91 sources was retained and synthesized (87 peer-reviewed publications and 4 regulatory framework documents). As a narrative review, selection was expert-guided and did not employ dual independent screening, formal risk-of-bias scoring, or predefined quantitative inclusion thresholds; the resulting corpus is therefore susceptible to selection bias and is intended to support conceptual synthesis rather than a comprehensive or fully reproducible enumeration of the literature.

## 3 Systemic biological age, epigenetic clocks, and skin-specific aging predictors

Biological age describes the biological state of an organism relative to chronological age and reflects cumulative variation in molecular damage, physiological reserve, immune–metabolic function, and stress-response capacity (5–8). DNA methylation-based epigenetic clocks represent the most extensively studied class of biological age biomarkers and demonstrate that age-associated methylation patterns can be quantified across tissues, cell types, and populations (6, 15–18).

Large-scale analyses indicate, however, that aging unfolds at different rates across organ systems within the same individual, and that accelerated aging of specific organ systems may predict morbidity and mortality more accurately than chronological age (19). This biological heterogeneity is conceptually central to precision esthetic longevity, because a systemic biological age estimate may diverge substantially from local cutaneous aging biology. As recently articulated by Furman et al. (20), the skin is best conceptualized as both a sentinel reflecting systemic biological aging and a potential modulator of organism-level inflammatory, immune, and neuroendocrine signaling — a bidirectional relationship that supports the integration of systemic and skin-derived biomarkers within a layered diagnostic framework.

Within the epigenetic clock family, three methodological generations can be distinguished. First-generation clocks (Horvath, Hannum) estimate chronological age from blood- or multi-tissue methylation signatures with high analytical accuracy, but their output remains age estimation rather than direct assessment of tissue function (6, 15). Second-generation clocks (PhenoAge, GrimAge) were trained against health- and mortality-related outcomes and align more closely with systemic inflammatory, metabolic, and physiological decline (16, 17). Pace-of-aging measures (DunedinPACE) estimate the rate of biological aging rather than a static age, and show improved test–retest reliability and associations with morbidity and disability (18). Despite their conceptual utility, all three classes were developed using blood-derived methylation data and were not designed to characterize cutaneous aging.

Several lines of evidence indicate that systemic epigenetic clocks underperform when applied to skin. A recent cross-tissue comparison demonstrated that clock outputs and inter-clock correlations differ substantially across tissues, underscoring the limited transferability of blood-derived clocks to skin and other organ systems (21). In addition, Kabacik et al. (22) reported that epigenetic age estimates do not necessarily correlate with the underlying cellular hallmarks of aging — including senescence burden, mitochondrial dysfunction, and proteostatic decline — indicating that methylation-derived age represents one dimension of biological aging rather than a unified surrogate for cellular function. These limitations have motivated the development of skin-specific predictors derived from epidermal tissue and designed explicitly to capture local cutaneous aging biology.

Several skin-relevant methylation predictors have now been described. Bormann et al. (23) provided early evidence that human skin aging is associated with reduced DNA methylation patterning and altered transcriptional connectivity within epidermal tissue. Horvath et al. (24) subsequently developed the Skin and Blood Clock, which uses 391 CpGs trained across fibroblasts, keratinocytes, buccal cells, endothelial cells, skin, blood, and saliva, achieving mean absolute errors of approximately 2.6 years in cultured fibroblasts and ~2.9 years on dermis/epidermis, outperforming the original pan-tissue clock in skin samples. Boroni et al. (25) extended this approach with MolClock, a skin-specific molecular methylation algorithm trained on 508 human skin samples (2,266 CpGs), designed to operate on epidermal methylation data, and subsequently used as a reference platform for evaluating skin-targeted interventions. Bienkowska et al. (26) introduced VisAgeX, the first second-generation skin-specific epigenetic clock, trained on methylation data from 378 female volunteers and designed to predict visible skin-aging phenotypes — including wrinkle grade, visual facial age, and visual age progression — rather than chronological age alone. Most recently, Menendez Vazquez et al. (27) introduced MitraSolo (single-CpG model) and MitraCluster (region-based model), both trained on the largest sequencing-based dataset of human epidermis published to date, achieving a mean absolute error of approximately 4 years on non-invasively collected tape-strip samples and outperforming the Horvath Pan-Tissue, Skin and Blood, Hannum, PhenoAge, and BS-Clock predictors on tape-strip-derived data; prediction accuracy was sex-agnostic and was not substantially affected by Fitzpatrick score, although the authors note that a broader dataset will be required to confirm phototype independence.

Beyond DNA methylation, complementary skin-aging biomarkers have been described in adjacent regulatory layers. Roig-Genoves et al. (28) developed a microRNA-based molecular clock that captures post-transcriptional aging dynamics in skin. Foucher et al. (29) further demonstrated in a cohort of 351 healthy women that clinical skin age, as assessed by dermatologists, can diverge from chronological age by up to 10 years, and that stratum corneum protein markers — including insulin-degrading enzyme (IDE), lipocalin-1 (LCN1), and transglutaminase-3 (TG3) — can distinguish accelerated from decelerated skin-aging trajectories, with higher IDE levels being protective against accelerated skin aging. These proteomic, microRNA-based, and methylation-based readouts are mechanistically complementary rather than redundant, and converge toward a multi-omic conception of cutaneous biological age (20, 27, 29). Table 2 summarizes these skin-specific epigenetic and molecular clocks, including their training cohorts, key performance characteristics, sample type, and references.

**Table 2:** Skin-specific epigenetic and molecular clocks for cutaneous biological age assessment.

Clock/predictor	Type/generation	Training cohort	Key feature	Sample type
Bormann et al. (23)	First epidermis-specific methylation evidence	Human epidermis; 108 white female donors, 18–79 y (450 k array)	Reduced methylation patterning and altered transcriptional connectivity in skin aging; foundation for later epidermal clocks	Biopsy
Skin and Blood Clock (Horvath) (24)	First-generation, multi-tissue	Fibroblasts, keratinocytes, buccal cells, endothelial cells, skin, blood, saliva (391 CpGs)	MAE ~ 2.6 y in cultured fibroblasts, ~2.9 y on dermis/epidermis (original); ~6.58 y on tape-strip epidermis per Mitra benchmark (27); outperforms pan-tissue clock on skin	Biopsy
MolClock (Boroni) (25)	Skin-specific molecular (DNAm)	508 human skin samples; 2,266 CpGs	First skin-specific molecular clock; MAE 1.94 y (training), RMSE 4.98 y (external); reference platform for screening interventions	Biopsy
VisAgeX (Bienkowska) (26)	Second-generation, skin-specific (phenotype)	378 female volunteers (SHIP)	Three clocks predicting wrinkle grade, visual facial age and visual age progression; VisAgeX MAE 4.67–6.17 y (LMR-based); not chronological-age based	Suction blister (volar forearm)
MitraSolo/MitraCluster (Menendez Vazquez) (27)	Sequencing-based (EM-seq), non-invasive (single-CpG/region)	*n* = 462 (training+test) from a 590-individual/817-sample tape-strip database	MitraCluster MAE 4.00 y, *R*^2^ = 0.89 (MitraSolo 4.09/0.88); sex-agnostic; Fitzpatrick-agnostic; intra-individual variation <2 y; robust at low sequencing depth	Tape-strip (non-invasive)
miRNA clock (Roig-Genoves) (28)	Post-transcriptional (microRNA), ML	64 healthy skin biopsies (4 public GEO datasets), 18–83 y; 1,856 miRNAs	First miRNA-based skin clock; ElasticNet MAE 10.89 y; classification up to 80.8% (SVC + SMOTE)	Public GEO small RNA-seq (biopsy)
Proteomic markers (Foucher) (29)	Stratum corneum proteomic	351 healthy women, 36–75 y	IDE protective against accelerated aging (*β* = −1.74); SC protein panel (IDE, LCN1, TG3) distinguishes accelerated vs. decelerated Age A/D	Tape-strip/D-squame

For each predictor, the methodological type/generation, training cohort, key performance or design feature, sample type, and reference are shown.

Reported mean absolute errors (MAE) are derived from the original publications and refer to the indicated tissue and validation context; values are not directly comparable across clocks due to differences in training tissue, platform, age range, and outcome (chronological age vs. visible phenotype vs. age acceleration/deceleration).

CpG, cytosine-phosphate-guanine dinucleotide; EM-seq, enzymatic methyl-sequencing; GEO, Gene Expression Omnibus; IDE, insulin-degrading enzyme; LCN1, lipocalin-1; LMR, low-methylated region; MAE, mean absolute error; SHIP, Study of Health in Pomerania; TG3, transglutaminase-3; Age A/D, age acceleration/deceleration.

Until recently, epigenetic age findings in skin were predominantly correlative. A 2023 epigenome-wide Mendelian randomization analysis identified 1,299 CpG sites with putative causal influence on perceived facial aging, providing the first large-scale evidence that specific methylation changes may contribute causally, rather than merely associatively, to visible esthetic phenotypes (30). At the same time, important methodological constraints persist. A longitudinal study tracking 64 individuals over 15 years found that most age-associated methylation markers did not follow uniform trajectories, demonstrating substantial inter-individual variability and underscoring the need for repeated-measures designs (31). Single-cell transcriptomic profiling of human skin has further revealed age-related loss of fibroblast priming and increased cellular heterogeneity within the dermis, indicating that bulk-tissue methylation profiles may obscure cell-type-specific aging signals (32). Most importantly, Koch et al. (33) recently proposed that somatic mutation accumulation, rather than active epigenetic regulation alone, may explain a substantial component of age-associated methylation drift, challenging the assumption that epigenetic clocks measure a purely regulatory aging process. While this hypothesis does not invalidate the predictive utility of methylation-based age estimates, it cautions against the interpretation of clock changes as direct evidence of “rejuvenation” or restored regulatory function (16, 22, 33).

In precision esthetic dermatology, systemic and skin-specific biological age biomarkers should therefore be interpreted as contextual and complementary measures rather than direct stand-alone markers of facial or cutaneous tissue age. First-generation clocks provide accurate age-estimation models, second-generation clocks and pace-of-aging measures are more closely aligned with systemic health risk and physiological decline, and skin-specific clocks may offer a more direct interface with local cutaneous aging biology (6, 15–18, 20–29). Their relevance to esthetic medicine lies in contextualizing systemic resilience, inflammatory tone, metabolic burden, vascular function, and potentially local skin-aging trajectories. However, these tools should not be treated as direct surrogates for dermal matrix integrity, pigmentation biology, barrier function, photoaging severity, treatment response, or biological rejuvenation unless validated for those specific esthetic contexts (16, 20, 22, 33). Early evidence linking systemic biological age clocks to skin biomechanical function in large community cohorts has begun to bridge molecular and functional layers of cutaneous aging (34), a connection that is examined in detail in the functional biomarker layer of this framework.

Clock outputs vary according to tissue source, assay platform, preprocessing method, training cohort, statistical model, and biological endpoint (6–8, 15–18, 21–29). Many models still lack validation across diverse phototypes and global populations, although emerging sequencing-based clocks such as MitraSolo and MitraCluster have been designed for broader, phototype-inclusive deployment, with initial evidence suggesting prediction accuracy is not substantially affected by Fitzpatrick score (27). Within the proposed five-layer diagnostic framework, biological age biomarkers serve as the first contextual layer; their responsible use requires defined context of use, analytical validity, clinical validity, reproducibility, phototype-inclusive validation, and clinically meaningful endpoints (7, 8, 35). At present, these biomarkers should inform research design and biological interpretation rather than guide treatment selection, procedural intensity, or claims of rejuvenation as stand-alone tools.

## 4 Skin-specific molecular biomarkers of esthetic aging

Skin-specific molecular biomarkers provide a local biological layer within precision esthetic longevity, complementing systemic biological age and longevity biomarkers. Systemic biomarkers reflect organism-level physiology, whereas skin-derived biomarkers may capture tissue-specific processes within the epidermis, dermis, vascular microenvironment, adnexal structures, and extracellular matrix (ECM). Visible skin aging emerges from local interactions among fibroblasts, keratinocytes, melanocytes, immune cells, endothelial cells, matrix proteins, barrier lipids, and environmental exposures, including ultraviolet radiation, pollution, smoking, and procedural injury (1–4, 36). Quan and Fisher (36) characterized age-associated ECM alterations as central to human skin aging, and Shin et al. (1) summarized molecular pathways linking dermal aging with collagen degradation, oxidative stress, inflammation, and impaired repair.

The ECM represents one of the most clinically relevant molecular biomarker domains in esthetic aging. Dermal collagen types I and III, elastin fibers, fibrillin-rich microfibrils, procollagen synthesis markers, matrix metalloproteinases (MMPs), and tissue inhibitors of metalloproteinases collectively reflect the balance between matrix synthesis, degradation, and remodeling (36–38). Age-associated collagen fragmentation alters fibroblast-matrix interactions, reduces mechanical tension within the dermis, impairs transforming growth factor beta signaling, and contributes to lower collagen production (36, 38). MMPs, particularly MMP-1 — the primary initiator of collagen fibril fragmentation — are constitutively elevated in photodamaged skin and contribute to a chronic pro-degradative microenvironment that progressively impairs dermal fibroblast function (39). Collagen breakdown also has implications beyond the dermal matrix itself, as proteolysis of collagen XVII (COL17A1) at the dermal–epidermal junction has been mechanistically linked to hair follicle stem cell loss and follicular miniaturization with age (40).

From a translational perspective, ECM biomarkers may help define the biological substrate upon which esthetic interventions act. Laser resurfacing, radiofrequency, microneedling, ultrasound-based tightening, and biostimulatory injectables depend on controlled injury, fibroblast activation, collagen synthesis, and matrix reorganization (1, 2, 36–38). A tissue environment characterized by fragmented collagen, increased MMP activity, reduced procollagen expression, or impaired fibroblast function may plausibly show weaker remodeling capacity or reduced durability of response. These parameters are generally assessed through biopsy-based histology, immunohistochemistry, gene-expression analysis, protein assays, or indirect structural surrogates such as dermal thickness, echogenicity, elasticity, and tissue recoil. Non-invasive imaging modalities such as multiphoton tomography further enable *in vivo* longitudinal monitoring of collagen and elastin organization (41). Their routine clinical value in esthetic practice remains inferential until validated against longitudinal treatment outcomes.

Between punch biopsy and superficial tape stripping lies a further translational gap in skin-specific molecular assessment. Punch biopsy reaches the dermal compartment—extracellular matrix, fibroblast populations, inflammatory infiltrates, vascular markers, and senescence-associated phenotypes—but its invasiveness, scarring risk, and limited patient acceptability make it unsuitable for the repeated sampling that longitudinal esthetic monitoring requires. Tape stripping is minimally invasive and repeatable, yet it captures only the stratum corneum and upper epidermis, leaving active dermal signals—collagen fragmentation, cytokine gradients, interstitial proteins, nucleic acids, and senescent-fibroblast markers—largely out of reach. Minimally invasive microneedle sampling and dermal interstitial fluid (dISF) extraction may bridge this gap. In human studies, microneedle-based approaches have sampled interstitial fluid with good tolerability, quantified protein biomarkers directly within it, and supported transcriptomic analysis from minimally invasive specimens (42–44); recent reviews position microneedle devices and interstitial-fluid biosensors as emerging platforms for repeated biochemical monitoring and personalized medicine (45, 46). In precision esthetic longevity, such tools could eventually track active dermal biology before and after lasers, microneedling, radiofrequency, injectables, or biostimulatory treatments—shifting molecular monitoring from the skin surface toward the dermal processes that actually drive treatment response. Realizing this will require standardization of sampling depth, analyte stability, assay platform, anatomical site, and safety, together with validation against defined esthetic outcomes.

Cellular senescence represents a second major molecular biomarker domain. Senescent cells accumulate in aging tissues and exhibit stable cell-cycle arrest together with secretion of inflammatory cytokines, chemokines, growth factors, proteases, and matrix-modifying factors, collectively termed the senescence-associated secretory phenotype (SASP) (47, 48). Campisi described SASP as a biologically active state capable of modifying tissue microenvironments (47). Principal biomarkers of cellular senescence in skin include p16INK4a, p21, senescence-associated beta-galactosidase, lamin B1 loss, DNA damage response markers, and SASP mediators such as interleukin-6, interleukin-1 beta, chemokines, growth factors, and MMPs (48). Senescence profiling can be performed through non-invasive tape stripping of the stratum corneum, enabling longitudinal quantification of SASP-related cytokines and matrix-degrading proteases by multiplex immunoassay platforms (49, 50). Waaijer et al. (51) linked p16INK4a-positive cell density in human skin to degraded elastic fiber morphology, deeper facial wrinkling, and older perceived facial age independent of chronological age. This finding directly links a measurable senescence biomarker to visible esthetic phenotypes of facial aging. p16INK4a nonetheless remains a context-dependent marker rather than an exclusive indicator of senescent fibroblasts.

In esthetic longevity, senescence biomarkers are relevant through their potential relationship to impaired repair, reduced fibroblast function, ECM degradation, persistent inflammation, and delayed tissue recovery (47, 48, 51). Recent pharmacological interventions targeting senescence have shown that the cutaneous senescence burden is modifiable *in vivo*: a short course of systemic senolytic therapy (dasatinib plus quercetin) reduced epidermal p16INK4a- and p21CIP1-positive cells within 11 days alongside circulating SASP factors, while topical rapamycin, a senomorphic mTOR inhibitor, reduced p16INK4a expression and increased dermal-epidermal junction collagen VII over 8 months, with concurrent improvement in clinical skin appearance (49). These represent proof-of-concept evidence that skin-derived senescence biomarkers are pharmacologically modifiable and clinically measurable. Within precision esthetic longevity, cutaneous senescence profiling may therefore serve as a diagnostic biomarker of an aged tissue phenotype and as a predictive biomarker of reduced procedural tolerance and altered tissue response (50). These applications remain investigational, and current clinical use is limited by sampling requirements, cell-type specificity, lack of standardized thresholds, and incomplete validation against esthetic outcomes.

Local inflammaging should be separated conceptually from systemic inflammatory burden. Systemic markers such as high-sensitivity C-reactive protein provide information about organism-level inflammatory tone, whereas local inflammatory biomarkers reflect signaling within the skin microenvironment. In aging and photoaged skin, persistent inflammatory activation may involve interleukin-6, interleukin-1 beta, tumor necrosis factor alpha, chemokines, MMPs, antimicrobial peptides, and SASP mediators (2, 47, 52). Franceschi et al. (52) framed inflammaging as an immune-metabolic feature of aging, while Pilkington et al. (53) characterized its cutaneous manifestations, including impaired barrier function, delayed wound healing, reduced immunosurveillance, matrix breakdown, and pigmentary instability (52, 53). Importantly, epidermal inflammatory signaling is not confined to the skin: experimental barrier disruption increases circulating inflammatory cytokines, suggesting that cutaneous inflammaging may contribute to systemic inflammatory burden (53).

Local inflammatory biomarkers may be assessed in research settings through skin biopsy, immunohistochemistry, transcriptomic profiling, protein assays, tape-stripping specimens, or cytokine profiling from skin-derived samples. In clinical esthetic practice, they are more often inferred from phenotype and procedural history, including erythema tendency, sensitivity, delayed healing, post-inflammatory hyperpigmentation risk, barrier instability, or exaggerated response to energy-based procedures. Their translational value lies in contextualizing tissue reactivity and recovery potential rather than serving as stand-alone predictors. Validated thresholds for esthetic treatment planning are not currently established (1, 2, 52).

Oxidative damage and redox imbalance represent another central biomarker domain in skin aging. The skin is continuously exposed to ultraviolet radiation, air pollution, visible light, inflammatory mediators, glycation-related stress, and mitochondrial reactive oxygen species (1, 3, 4, 54). Oxidative injury can affect lipids, proteins, DNA, mitochondria, and ECM components, contributing to photoaging, pigmentation irregularity, barrier dysfunction, fibroblast impairment, MMP activation, reduced collagen synthesis, and delayed wound repair (54). Rittie and Fisher (3) described natural and sun-induced skin aging as processes linked to ultraviolet-mediated molecular injury, Krutmann et al. (4) positioned environmental exposure as a central driver of the skin-aging exposome, and Gu et al. (54) summarized the central role of reactive oxygen species and autophagy in skin aging, linking oxidative stress with cellular senescence, MAPK and NF-kappa B signaling, MMP induction, and ECM degradation.

Candidate oxidative damage and redox biomarkers include F2-isoprostanes, malondialdehyde, thiobarbituric acid reactive substances, 8-hydroxy-2′-deoxyguanosine, protein carbonyls, advanced oxidation protein products, reduced and oxidized glutathione, the glutathione ratio, superoxide dismutase, glutathione peroxidase, catalase activity, and total antioxidant capacity. These markers differ in biological meaning: F2-isoprostanes are relatively robust markers of lipid peroxidation; malondialdehyde and thiobarbituric acid reactive substances are widely used but less specific; 8-hydroxy-2′-deoxyguanosine reflects oxidative DNA damage; and glutathione or antioxidant enzyme measurements reflect redox defense rather than direct tissue injury. In routine esthetic practice, oxidative burden remains largely indirect and phenotype-based (1, 3, 4).

Glycation and advanced glycation end products (AGEs) form a mechanistically distinct but interacting biomarker domain. AGEs accumulate in long-lived proteins such as collagen and elastin, promoting cross-linking, stiffness, altered matrix turnover, oxidative stress, and receptor for advanced glycation end products signaling (55). Gkogkolou and Bohm reviewed the role of AGEs in skin aging, emphasizing dermal stiffness, loss of elasticity, inflammation, and impaired tissue quality (55). These mechanisms are relevant to esthetic tissue behavior through their potential effects on elasticity, recoil, vascular function, and remodeling capacity.

Glycation-related biomarkers may include metabolic drivers such as glucose, glycated hemoglobin, insulin resistance indices, circulating AGE species such as carboxymethyllysine, carboxyethyllysine, pentosidine, methylglyoxal-derived hydroimidazolone, reactive dicarbonyl precursors, and non-invasive skin autofluorescence (55, 56). Skin autofluorescence provides a practical surrogate for fluorescent AGE accumulation and has been validated in non-pigmented skin against skin-biopsy-derived AGE accumulation (56). In esthetic dermatology, its value remains investigational. Results may be influenced by pigmentation, smoking, renal function, anatomical site, metabolic state, and device methodology. Lee et al. (57) reported that AGEs may promote melanogenesis through receptor for advanced glycation end products signaling, linking glycation biology with pigmentary pathways relevant to photoaging.

Epidermal barrier biomarkers provide a more accessible molecular and functional interface between skin biology and clinical phenotype. Barrier integrity depends on stratum corneum lipid organization, ceramides, cholesterol, free fatty acids, natural moisturizing factor, filaggrin-derived products, corneocyte maturation, tight junctions, acid mantle regulation, and local inflammatory signaling (58). de Boer et al. (58) reviewed epidermal biomarkers associated with barrier function and integrity, highlighting molecular, morphological, and biophysical markers, many obtained from stratum corneum tape-stripping methods. With aging, reduced epidermal lipid synthesis, altered stratum corneum pH, and delayed barrier recovery kinetics collectively contribute to functional barrier decline; in a classic study, young subjects achieved 50% barrier recovery at 24 h following disruption compared with only 15% in aged subjects (49, 59).

For esthetic longevity, barrier biomarkers are clinically relevant through their relationship to sensitivity, topical tolerance, inflammatory reactivity, post-procedural erythema, re-epithelialization, and recovery after resurfacing, peeling, microneedling, radiofrequency, and laser-based interventions. Transepidermal water loss, corneometry, and skin surface pH are functional measures discussed later in the framework, while molecular barrier markers include ceramide profiles, natural moisturizing factor, filaggrin-related breakdown products, corneocyte maturity markers, lipidomic signatures, and inflammatory mediators obtained through tape stripping or tissue-based methods. Direct clinical use remains limited by variability in sampling methods, device platforms, environmental conditions, and absence of esthetic-specific thresholds (58).

Genetic determinants represent a complementary biomarker layer that links inherited biological architecture to skin aging trajectories, photodamage susceptibility, pigmentation, elasticity, barrier function, and tissue repair capacity (60, 61). Whereas epigenetic biomarkers capture dynamic, environmentally responsive modifications of gene expression, genetic biomarkers reflect inherited variation that may shape interindividual differences in baseline aging susceptibility and treatment response (61). Geusens and Haykal estimated that genetic factors may explain up to 60% of the variability in skin aging in specific populations, while emphasizing that this figure reflects heritability under controlled conditions and does not negate the substantial influence of lifestyle and environmental exposure (61).

Several genes have been associated with discrete aspects of skin aging biology. Elastin (ELN) contributes to dermal elasticity and mechanical recoil, and polymorphisms affecting elastin expression and turnover may influence interindividual variation in elastic fiber integrity, solar elastosis, and biomechanical aging (61). Filaggrin (FLG) is essential to corneocyte maturation, natural moisturizing factor generation, and barrier integrity, and loss-of-function variants in FLG have been associated with impaired barrier function and increased transepidermal water loss (61). Melanocortin 1 receptor (MC1R) influences pigmentation, ultraviolet response, and phototype-related photoaging susceptibility, representing one of the most extensively characterized determinants of pigmentary aging biology, while MMP-1 promoter polymorphisms have been associated with collagen-degrading activity and susceptibility to dermal aging (61).

Naharro-Rodriguez et al. (60) emphasized that many genes implicated in skin aging exhibit pleiotropy, with single-nucleotide polymorphisms (SNPs) predominantly located within a small group of approximately 44 central pleiotropic genes and 32 genes associated with skin pigmentation, many of which are clustered in chromosomal band 16q24.3. The pleiotropic architecture of these loci suggests that genes traditionally linked to pigmentation may also contribute to wrinkle formation, loss of elasticity, and other visible aging phenotypes (60). Transcriptomic analyses further indicate that most age-associated genes are differentially expressed with age, supporting a functional link between gene expression profiles and observable aging trajectories (60). Familial aggregation studies suggest that individuals with relatives exhibiting slower aging are more likely to experience delayed cutaneous aging themselves, reinforcing the inherited component of skin aging biology (60).

Polygenic risk scores aggregate the effects of multiple variants and represent an evolving approach to stratifying individuals according to inherited aging trajectories (61). Haykal and Flament emphasized that aging is a heterogeneous process shaped by genetic, environmental, and lifestyle factors, and that precision approaches incorporating multi-omic profiling, longitudinal monitoring, and computational modeling may enable classification of individuals according to dominant biological mechanisms, including inflammaging-dominant, matrix-degradation-dominant, and senescence-dominant subtypes (49). Within precision esthetic longevity, integrated assessment of genetic and epigenetic biomarkers may offer the most direct interface between inherited biological architecture and modifiable aging biology (49, 60, 61).

Translation of genetic biomarkers into clinical esthetic practice nonetheless remains preliminary. Most published associations are derived from observational cohorts in restricted populations, with limited validation across phototypes, ethnicities, anatomical sites, and esthetic outcomes (60, 61). Reported heritability estimates reflect population-level variance rather than individual treatment response, regenerative capacity, or durability of esthetic intervention (61). At present, genetic biomarkers in esthetic medicine should therefore be regarded as research-stage tools for stratification, prevention, and mechanistic interpretation, requiring prospective validation against clinically meaningful esthetic endpoints before adoption as treatment-selection instruments (60, 61).

Taken together, skin-specific molecular biomarkers define the local biological substrate of esthetic aging. ECM remodeling, senescence, local inflammation, oxidative damage, glycation, barrier integrity, and genetic susceptibility represent interconnected domains rather than isolated pathways. Their greatest current value lies in research stratification, mechanistic interpretation, and future biomarker-informed clinical studies. In routine practice, most of these markers are inferred through phenotype, imaging, functional measures, systemic biomarkers, and procedural response rather than measured directly. Responsible translation requires analytical validity, skin-specific clinical validity, reproducibility, minimally invasive sampling strategies, phototype-inclusive validation, and longitudinal correlation with meaningful esthetic outcomes (1–4, 36–38, 47–49, 51, 52, 54–58, 60, 61).

## 5 Functional tissue biomarkers and functional correlates of regenerative capacity

Functional tissue biomarkers describe how skin behaves under baseline and stress conditions, rather than only what it contains or how it appears. In precision esthetic longevity, this layer links molecular skin aging with clinically observable tissue performance (1, 36, 62–68). Skin quality and response to intervention depend on barrier competence, hydration state, biomechanical behavior, microvascular support, inflammatory resolution, and repair kinetics (62–68). Functional measurements therefore provide dynamic information that cannot be obtained from molecular profiling, static imaging, or visual grading alone (62–68).

Barrier function is one of the most established functional domains in skin assessment. Transepidermal water loss (TEWL) measures passive water flux through the stratum corneum and is widely used as a non-invasive indicator of epidermal barrier integrity (62, 63). Rogiers, through the European Group on Efficacy Measurement and Evaluation of Cosmetics and Other Products (EEMCO), provided methodological guidance for TEWL assessment in cosmetic sciences, emphasizing environmental control, anatomical-site consistency, acclimatization, instrumentation, and standardized interpretation (62).

In esthetic dermatology, increased TEWL may indicate impaired barrier integrity and may be relevant to sensitivity, irritant reactivity, prolonged erythema, delayed re-epithelialization, and reduced tolerance to resurfacing, peeling, microneedling, retinoids, acids, and energy-based procedures. Beyond baseline TEWL, dynamic assessment of barrier recovery kinetics following controlled disruption may provide a more discriminating functional readout, as aged skin demonstrates markedly delayed barrier recovery despite preserved baseline values (49). TEWL should be interpreted as a functional biomarker of epidermal barrier status, not as a direct measure of dermal aging, collagen remodeling, or regenerative capacity (62, 63).

Stratum corneum hydration provides a complementary functional measure. Corneometry estimates superficial water content using electrical properties of the stratum corneum and is widely used in dermatologic and cosmetic research. Berardesca described the biophysical basis, sources of variability, and methodological limitations of electrical hydration measurements (64), while the revised EEMCO guidance reaffirmed the importance of standardized measurement conditions for *in vivo* assessment of water in the skin (63).

Hydration influences optical smoothness, roughness, desquamation, tactile texture, and tolerance to topical agents. In esthetic longevity, low stratum corneum hydration may contribute to rough texture and reduced procedural tolerance. Corneometry primarily reflects superficial hydration and should not be interpreted as a standalone indicator of deeper dermal quality, extracellular matrix (ECM) integrity, or tissue resilience (63, 64).

Skin elasticity is a key functional biomarker of biomechanical tissue behavior. Elasticity reflects the integrated contribution of the ECM, collagen organization, elastin integrity, hydration, dermal thickness, and mechanical coupling between skin and underlying tissues. Cutometry is the most widely used suction-based method for quantifying viscoelastic skin properties. The Cutometer applies controlled negative pressure and measures deformation and recovery kinetics, generating parameters related to distensibility, gross elasticity, net elasticity, and biological elasticity (65). Complementary biomechanical tools include the Reviscometer, which captures anisotropic viscoelastic properties reflecting directional fiber organization, and the Ballistometer, which assesses dynamic mechanical resilience (49).

Ryu et al. (65) reported age- and region-related differences in skin elasticity measured with Cutometer, supporting the relevance of biomechanical assessment in aging skin research. In esthetic practice, reduced elasticity may inform expectations regarding tightening, lifting, collagen stimulation, and recovery. Interpretation remains protocol-dependent and influenced by anatomical site, hydration, probe pressure, device settings, age, photodamage, and measurement conditions (65). Recent evidence has begun to link biomechanical skin properties directly to systemic biological aging: in the INSPIRE-T cohort of 441 adults aged 20 to 93, accelerated inflammatory aging was associated with reduced Cutometer-measured skin elasticity and viscoelasticity, and poorer skin biomechanical performance correlated with lower intrinsic capacity and faster longitudinal functional decline (34).

Microcirculation represents a functional domain closely linked to repair and tissue resilience. Cutaneous perfusion supports oxygen delivery, nutrient transport, immune surveillance, inflammatory resolution, thermoregulation, and wound healing. Laser Doppler flowmetry, laser Doppler imaging, and laser speckle contrast imaging are non-invasive approaches used to assess skin blood flow and microvascular reactivity (66–68).

Cracowski et al. (66) reviewed methodological issues in assessing skin microvascular endothelial function, and Roustit and Cracowski (67) summarized non-invasive methods for evaluating human skin microvascular function. Laser speckle contrast imaging provides spatial and temporal assessment of relative blood flow and has been used to study microvascular reactivity (68). In esthetic longevity, impaired microcirculation may contribute to delayed healing, prolonged inflammation, vascular instability, and weaker functional recovery, particularly in patients with smoking exposure, metabolic dysfunction, inflammatory disease, or advanced photodamage.

Wound healing capacity represents an integrative clinical-functional endpoint rather than a single biomarker. It reflects coordinated barrier repair, inflammatory resolution, keratinocyte migration, fibroblast activation, angiogenesis, ECM deposition, and remodeling. Esthetic interventions such as fractional lasers, radiofrequency, microneedling, chemical peeling, ablative resurfacing, and biostimulatory injectables intentionally induce controlled injury to activate repair pathways (62–68).

Clinical healing kinetics, including time to re-epithelialization, duration of erythema, edema, crusting, discomfort, pigmentary instability, and fibrosis, may provide practical indicators of tissue resilience. Direct molecular measurement of wound repair remains largely research-based. Experimentally, re-epithelialization rate can be quantified by serial planimetric imaging and suction-blister protocols enable sampling of local growth factors and inflammatory mediators, with the time required to reconstitute a competent stratum corneum more than doubling in elderly compared with young adults (49). In clinical translation, previous procedural response, barrier testing, perfusion assessment, standardized photography, and structured follow-up provide more feasible functional correlates of regenerative capacity (62–68).

The translational value of functional biomarkers lies in their ability to connect biological plausibility with observable tissue behavior. A patient with impaired barrier function, low hydration, reduced elasticity, and suboptimal perfusion may demonstrate a different recovery profile from a patient with the same visible aging phenotype but preserved functional tissue performance. Functional assessment may therefore support pre-procedural optimization, procedural intensity selection, staged treatment planning, and longitudinal monitoring. Current evidence does not support rigid functional thresholds for esthetic treatment selection (62–68).

Functional tissue biomarkers occupy a central position in the proposed multilayer framework. They translate skin biology into measurable tissue performance and help define functional correlates of regenerative capacity in clinically observable terms. Responsible use requires standardized acquisition, environmental control, test–retest reliability, anatomical consistency, and validation against meaningful esthetic outcomes. At present, TEWL, corneometry, cutometry, and microvascular assessment are best regarded as complementary tools for research and selected clinical contexts rather than universal decision-making instruments. Their future value will depend on longitudinal studies linking functional measurements to recovery kinetics, treatment response, durability of improvement, and patient-relevant outcomes (62–68).

## 6 Imaging-based and morphometric diagnostics

Imaging-based diagnostics provide an objective extension of clinical skin assessment by translating visible, optical, structural, and morphometric features into measurable parameters. In precision esthetic longevity, imaging does not replace molecular or functional biomarkers; it defines an additional diagnostic layer that captures how biological aging processes are expressed at the level of skin surface, dermal architecture, vascular and pigmentary patterns, facial geometry, and longitudinal treatment response. The principal value of imaging lies in standardized documentation, objective comparison over time, and quantitative assessment of features that are difficult to grade reproducibly by visual inspection alone (69–76).

Standardized clinical photography remains the foundation of esthetic documentation. Controlled lighting, camera settings, patient positioning, facial expression, distance, background, and timing are essential for reliable comparison across visits (69). Photography captures global phenotype, pigmentation, erythema, contour, surface texture, wrinkle pattern, and visible response to treatment. Its main limitation is that it remains surface-based and acquisition-dependent. Parsa et al. (69) reviewed current and future photography techniques in esthetic surgery and emphasized the evolution from two-dimensional photographic documentation toward three-dimensional and quantitative imaging methods. Standardized photography should therefore be regarded as the baseline imaging layer rather than a complete diagnostic system.

Multispectral and surface imaging systems extend photographic assessment by analyzing wavelength-specific features related to pigmentation, erythema, vascularity, ultraviolet-associated change, pores, wrinkles, and texture. VISIA-type systems are widely used in cosmetic dermatology for standardized facial imaging and longitudinal monitoring. Zawodny et al. (70) used the VISIA Skin Analysis System to evaluate reduction of pigmented and vascular facial lesions after 532-nm laser treatment, supporting its utility for selected longitudinal cosmetic endpoints. These systems are valuable when the measurement task is clearly defined, such as tracking pigmentary or vascular change over time. Their outputs remain device-specific and should not be interpreted as universal biological biomarkers of skin age, inflammation, or treatment response without validation for that specific claim.

Three-dimensional imaging and stereophotogrammetry quantify facial geometry, surface displacement, asymmetry, volume distribution, contour change, and topographic variation. These measurements are particularly relevant to esthetic dermatology and surgery, where visible aging reflects not only epidermal or dermal changes but also fat redistribution, ligamentous laxity, skeletal remodeling, soft-tissue descent, and procedural volume alteration. Three-dimensional imaging can improve objective documentation of fillers, surgery, tightening procedures, contour change, and surface topography, provided that image registration, facial expression, positioning, and acquisition protocols are standardized. Machado et al. (71) reported that three-dimensional stereophotogrammetry can quantify topographical skin-surface changes following treatment, supporting its role as an objective outcome-assessment tool.

Structured-light and surface-topography platforms provide more focused assessment of wrinkle depth, roughness, texture, scars, and microtopographic change. These systems are useful for resurfacing studies and interventions targeting surface quality rather than volume. Their diagnostic meaning is primarily morphometric: they quantify surface change, not the molecular mechanism producing that change. A reduction in wrinkle depth may reflect hydration, edema, resurfacing, extracellular matrix remodeling, or optical smoothing depending on timing and intervention. Morphometric findings should therefore be interpreted alongside clinical context, functional measures, and, where available, molecular or ultrasound-based indicators of dermal remodeling (71).

High-frequency ultrasound is one of the most relevant imaging modalities for precision esthetic longevity, as it provides non-invasive assessment below the visible surface. High-frequency ultrasound can measure skin thickness, dermal echogenicity, subepidermal low-echogenic bands, filler localization, edema, fibrosis, soft-tissue planes, and selected procedure-related changes (72, 73, 76). Levy et al. (72) reviewed high-frequency ultrasound in dermatology and noted its value in evaluating skin thickness and echogenicity, while Czajkowska et al. (73) described applications in esthetic medicine, including dermal thickness assessment, filler visualization, and treatment monitoring. Ultrasound is particularly important in injectable esthetics, where it may support safety, complication evaluation, and localization of hyaluronic acid filler deposits. Its interpretation remains operator-dependent and influenced by probe frequency, pressure, gain settings, anatomical site, and scanning plane (72, 73, 76).

Optical coherence tomography and reflectance confocal microscopy provide higher-resolution visualization of superficial skin architecture. Optical coherence tomography allows non-invasive cross-sectional imaging of epidermal and superficial dermal structures and has been applied to chronological aging, photoaging, and photodamage research (74). Mamalis et al. (74) described optical coherence tomography as useful for visualizing dermal collagen-related changes during aging and photodamage. Reflectance confocal microscopy provides near-cellular resolution of epidermal and superficial dermal microarchitecture and has been used to evaluate photoaging-associated features. Guida et al. (75) summarized reflectance confocal microscopy features of aging skin and skin cancer, supporting its relevance to dermatologic aging research. These modalities offer greater microstructural detail than conventional photography, yet their penetration depth, field size, acquisition time, and need for expertise limit routine integration into esthetic practice.

Advanced high-resolution optical modalities provide research-grade quantification of dermal fiber architecture. Multiphoton microscopy exploits second-harmonic generation from fibrillar collagen and two-photon excited autofluorescence from elastin, enabling simultaneous *in vivo* assessment of both fiber populations; from these signals, the SHG-to-autofluorescence aging index of dermis (SAAID) was derived as a validated quantitative metric that decreases progressively with age and photoaging severity (77, 78). More recently, line-field confocal optical coherence tomography (LC-OCT) has emerged as a volumetric modality achieving near-isotropic resolution of approximately one micrometer with penetration depths up to 500 micrometers, allowing real-time three-dimensional reconstruction of dermal architecture with precise localization relative to the dermal–epidermal junction (79).

LC-OCT is best understood not as a variant of conventional OCT but as a hybrid modality that resolves the long-standing trade-off between the cellular resolution of reflectance confocal microscopy (RCM) and the depth of optical coherence tomography (OCT). Line-field illumination combined with the optical principles of both techniques allows LC-OCT to generate vertical, horizontal, and three-dimensional reconstructions in real time at near-cellular resolution (~1 μm) to depths of approximately 500 μm (80). This combination makes it especially suited to precision esthetic longevity, enabling repeated, non-invasive assessment of epidermal thickness, dermal–epidermal junction morphology, superficial dermal architecture, and age-related microstructural change.

Its value is increasingly supported by quantitative studies. Ayadh et al. (81) mapped age- and body-site-dependent variation in human skin morphology *in vivo*, establishing LC-OCT as a tool for quantifying structural aging phenotypes. Coupling three-dimensional LC-OCT with automated image analysis, Bonnier et al. (82) derived facial aging biomarkers—layer thickness, dermal–epidermal junction undulation, cell surface density, nuclear volume, and cellular-network atypia—in 100 healthy women aged 20–70 years, and Assi et al. (83) trained a deep-learning model that predicted chronological age from superficial dermal features, drawing chiefly on collagen-network organization beneath the dermal–epidermal junction. Together, these findings position LC-OCT as a reference hybrid modality for real-time, depth-resolved, non-invasive assessment of cutaneous aging architecture, and as a natural anchor for multimodal validation studies linking imaging-derived microstructure to biological and clinical outcomes.

Imaging-based diagnostics should be interpreted according to the anatomical depth and biological domain captured by each modality. Standardized photography and multispectral imaging primarily assess surface and optical phenotype. Three-dimensional imaging quantifies geometry and contour. Surface-topography systems assess microrelief and texture. High-frequency ultrasound evaluates dermal and subcutaneous structure. Optical coherence tomography and reflectance confocal microscopy visualize superficial microarchitecture. No single modality captures the full biology of skin aging. Imaging outputs are therefore most informative when matched to a defined diagnostic question and interpreted within a multilayer model that includes systemic context, skin-specific molecular biology, functional tissue behavior, and clinical phenotype (69–76).

The major translational challenge is validation. Many imaging systems generate numerical scores for pigmentation, redness, pores, wrinkles, texture, skin age, dermal thickness, echogenicity, contour, or volume. Numerical output alone does not establish biomarker status. A device-derived measure requires evidence of analytical reliability, test–retest reproducibility, standardized acquisition, sensitivity to clinically meaningful change, and validation in the intended population and use case. Phototype diversity is especially important for pigmentary, vascular, erythema, and optical measurements. Proprietary algorithms and device-specific scoring further limit comparability across platforms (7–11).

Within the proposed framework, imaging-based and morphometric diagnostics occupy an intermediate layer between molecular biology and visible clinical phenotype. Their strongest current role lies in baseline documentation, treatment planning, complication assessment, longitudinal monitoring, and objective outcome measurement. Their future value in precision esthetic longevity will depend on integration with molecular biomarkers, functional tissue measurements, artificial intelligence-derived digital phenotyping, and validated clinical endpoints. Imaging can quantify structural and optical manifestations of aging, but it should not be used as a stand-alone surrogate for biological rejuvenation, molecular repair, or treatment efficacy without longitudinal validation (69–76).

## 7 AI-derived digital phenotyping and digital biomarkers

Artificial intelligence (AI)-derived digital phenotyping represents the fifth diagnostic layer in the proposed framework, translating visual, imaging, and clinical data into quantitative descriptors of skin and facial aging. In precision esthetic longevity, AI should be interpreted as an analytical extension of clinical assessment rather than as an independent biological measurement. Its principal value lies in standardized phenotypic evaluation, improved reproducibility, longitudinal comparison, and potential integration of multimodal data. Rajkomar et al. (84) described machine learning in medicine as a method for identifying patterns in complex clinical datasets, while Topol (85) emphasized the convergence of human expertise and computational analysis in high-performance medicine.

A digital phenotype refers to the computational representation of observable skin and facial features (84–87). The digital phenotype commonly includes quantified features such as wrinkle depth and distribution, texture, pores, erythema, pigmentation, vascular patterns, acne or scar morphology, surface roughness, facial asymmetry, contour change, volume distribution, and apparent age (86, 87). These outputs can improve objectivity compared with unstructured visual assessment, yet they remain phenotypic descriptors unless linked to a validated biological or clinical endpoint (7–11). A wrinkle score, pigmentation index, redness score, or skin-age estimate can support documentation and follow-up, but it should not be interpreted as a direct measure of collagen degradation, melanocyte activity, vascular biology, inflammation, or biological rejuvenation without validation for that specific context of use (7–11, 86–88).

A clear conceptual separation is required between a digital measurement, a digital endpoint, and a validated digital biomarker (7–11). A digital measurement is a quantitative output generated from an image, device, or computational platform. A digital endpoint is a measurement used to evaluate change over time or response to an intervention. A validated digital biomarker requires evidence that the measurement is analytically reliable, clinically meaningful, and relevant to a defined biological or clinical state (7–11). In esthetic longevity, many AI-derived outputs currently fall closer to quantitative phenotyping than validated biomarker status. Their interpretation therefore depends on acquisition standardization, reproducibility, ground-truth definition, external validation, and clinically meaningful thresholds (7–11, 86–89).

AI-based image analysis has already demonstrated value in dermatology, particularly in lesion classification and diagnostic support. Esteva et al. (90) showed that a deep neural network trained on clinical images could classify skin cancer with dermatologist-level performance under defined experimental conditions. This work established a major foundation for dermatologic AI, yet translation to skin-aging assessment requires a different evidentiary framework. Aging-related features are continuous, multifactorial, and influenced by lighting, image quality, facial expression, cosmetic use, phototype, prior procedures, and environmental exposure. These variables make acquisition standardization, population diversity, and outcome definition essential for valid interpretation.

Automated facial and skin-aging analysis has expanded through standardized imaging platforms, selfie-based algorithms, and commercial skin-analysis systems. Flament et al. (86) demonstrated that algorithm-based analysis can quantify facial aging signs from selfie images in 1,041 women in the United States across ages, ancestries, and phototypes. In a large-scale AI analysis of 544,603 women, including 465,587 European women and 79,016 Chinese women, Flament et al. (87) further reported population-specific differences in visible facial skin-aging patterns. Together, these studies support the feasibility of algorithmic assessment of visible aging features in American, European, and Asian female populations, while emphasizing that clinical interpretation depends on training-data quality, ground-truth validity, imaging conditions, population diversity, phototype performance, and the intended use of the output.

Ground truth is a central limitation in AI-derived esthetic assessment. An algorithm trained to reproduce expert wrinkle grading models expert visual assessment rather than dermal biology. An algorithm trained on apparent age models perceived age rather than local skin biological age. A model trained on standardized clinical photography is not automatically valid for consumer selfies, and a model trained primarily on lighter phototypes cannot be assumed to perform equivalently across darker phototypes. These issues are particularly relevant for pigmentation, erythema, vascularity, texture, and apparent-age outputs, all of which are highly sensitive to image acquisition and population characteristics (86, 87).

AI-derived facial age is particularly relevant to esthetic longevity, although its interpretation requires precision. Bontempi et al. (88) developed and validated FaceAge, a deep-learning system estimating biological age from facial photographs, and reported prognostic value in non-esthetic medical populations. This supports the concept that facial appearance can contain biologically meaningful information related to systemic health and aging. It does not establish AI-derived facial age as a validated esthetic treatment-planning tool, a direct measure of local cutaneous aging, or evidence of rejuvenation after intervention. In esthetic longevity, facial-age models should therefore be regarded as hypothesis-generating and contextual unless validated against skin-specific biology, longitudinal outcomes, and treatment-response endpoints.

Commercial skin-analysis platforms add a further interpretive challenge. Many systems quantify pigmentation, vascularity, pores, wrinkles, texture, ultraviolet-associated change, redness, skin quality, or apparent age. Some platforms have peer-reviewed support for specific measurement tasks, particularly standardized documentation or longitudinal monitoring of pigmentary and vascular changes. Numerical output, market availability, and visually persuasive scores do not equal biomarker validation. A platform can be useful for reproducible documentation while remaining unvalidated for diagnosis, biological interpretation, treatment selection, or outcome prediction. Proprietary algorithms further limit independent evaluation, cross-platform comparison, and transparency regarding training datasets, phototype performance, ground truth, and clinically meaningful thresholds (86, 87, 89).

Validation standards should correspond to the clinical claim. Tools used for documentation or phenotypic quantification require evidence of reproducibility, acquisition standardization, and stability across common sources of variation (9–11). Tools proposed for risk stratification, diagnosis, treatment selection, outcome prediction, or biological-age claims require stronger evidence, including external validation, calibration, bias assessment, and clinical utility. CONSORT-AI and SPIRIT-AI were developed to improve transparent reporting of clinical trials and protocols involving AI interventions, emphasizing intended use, data quality, human-AI interaction, error analysis, and workflow integration (12, 13). FDA guidance on AI-enabled device software functions further emphasizes lifecycle management, risk management, and documentation for marketing submissions (14).

Bias and generalizability are central concerns in skin and facial AI. Daneshjou et al. (91) reported disparities in dermatology AI performance on a diverse clinical image set, reinforcing the need for validation across skin tones and disease presentations. The European Academy of Dermatology and Venereology AI Task Force has also emphasized transparency, performance evaluation, and responsible implementation of AI-assisted smartphone and web-based dermatology services (89). In esthetic longevity, biased performance could affect assessment of pigmentation, erythema, vascularity, texture, apparent age, and treatment response. Phototype-inclusive validation is therefore a core scientific requirement for equitable and reliable digital phenotyping.

The most promising role for AI in precision esthetic longevity is multimodal integration rather than isolated image scoring. Future systems could combine standardized photography, multispectral imaging, three-dimensional morphometry, high-frequency ultrasound, optical coherence tomography, functional measurements, molecular biomarkers, systemic biological-age indicators, and clinical outcomes. Such models could support longitudinal phenotyping, identification of dominant aging patterns, treatment-response monitoring, and research stratification. Current evidence does not yet demonstrate that AI-guided esthetic treatment selection improves outcomes across procedures in prospective trials (84, 85).

Within the proposed framework, AI-derived digital phenotyping should be positioned as a quantitative and integrative diagnostic layer. Its strongest current applications are standardized phenotyping, objective documentation, longitudinal monitoring, and research-based integration of multimodal data. Its future clinical value will depend on transparent methodology, defined context of use, external validation, phototype-diverse datasets, bias assessment, clinically meaningful endpoints, and evidence of utility beyond standard expert assessment. AI can support the transition toward mechanism-informed and longitudinal evaluation, but it should not be used to claim biological rejuvenation, predict treatment response, or replace clinical expertise without appropriate validation (12–14, 84, 85, 89, 91).

## 8 A multilayer translational diagnostic framework for precision esthetic longevity

Based on the reviewed evidence, we propose a five-layer translational diagnostic framework for precision esthetic longevity. The framework is not intended to function as a single score, fixed biomarker panel, or automated treatment algorithm. Its purpose is to organize complementary diagnostic domains into a structured interpretive model that links systemic aging biology, local skin molecular processes, tissue function, structural imaging, and digital phenotyping with clinically meaningful assessment.

The first layer consists of systemic biological age and longevity biomarkers. Epigenetic clocks, pace-of-aging measures, inflammatory and metabolic biomarkers, immune-aging signatures, and composite clinical biomarker indices provide information about organism-level physiology. Their relevance to esthetic dermatology lies in contextualizing systemic resilience, inflammatory burden, metabolic stress, vascular risk, and repair capacity. These biomarkers may inform the biological background in which skin aging and procedural recovery occur, but they should not be interpreted as direct measures of facial skin age, dermal quality, or esthetic rejuvenation (6, 15–18, 24, 26, 27).

The second layer includes skin-specific molecular and epigenetic biomarkers. This layer captures local biological processes within the epidermis, dermis, vascular microenvironment, and extracellular matrix, including skin-specific DNA methylation patterns, ECM remodeling, cellular senescence, local inflammation, oxidative damage, advanced glycation end products, barrier-related markers, and genetic determinants of aging susceptibility. Compared with systemic biomarkers, this layer is more directly related to cutaneous aging biology, yet most markers remain research-level and require validation against clinically meaningful esthetic outcomes (1–4, 36–38, 47, 51, 52, 55–58).

The third layer consists of functional tissue biomarkers. These measurements evaluate tissue behavior rather than molecular composition or visible appearance alone. Transepidermal water loss, corneometry, skin surface pH, elasticity assessment, cutometry, microcirculatory imaging, and healing kinetics provide information about barrier competence, hydration, biomechanical integrity, perfusion, inflammatory resolution, and functional correlates of regenerative capacity. Within the framework, functional biomarkers serve as an intermediate layer between molecular aging mechanisms and clinical response, helping to explain why patients with similar visible phenotypes may differ in procedural tolerance, recovery dynamics, and remodeling capacity (62–68).

The fourth layer includes imaging-based and morphometric diagnostics. Imaging modalities quantify the structural, optical, spatial, and microstructural expression of skin and facial aging across different tissue depths. Standardized photography, multispectral imaging, three-dimensional morphometry, surface topography, high-frequency ultrasound, optical coherence tomography, reflectance confocal microscopy, and multiphoton-based modalities can document baseline phenotype, monitor longitudinal change, assess dermal and subcutaneous architecture, evaluate filler distribution, and support complication assessment. The clinical and research relevance of these modalities is supported by multiple studies across dermatology, cosmetic science, and esthetic medicine. Their principal limitation is that most imaging outputs remain descriptive rather than mechanistic unless interpreted alongside molecular, functional, and clinical data (69–76).

The fifth layer is artificial intelligence-derived digital phenotyping. Digital phenotyping converts visible and imaging-derived features into quantitative computational descriptors, including wrinkle distribution, pigmentation, erythema, texture, pores, vascular patterns, facial contour, asymmetry, and apparent age. Its value lies in standardization, reproducibility, longitudinal comparison, and future multimodal integration. However, artificial intelligence-derived outputs should not be treated as biological biomarkers unless they demonstrate analytical reliability, external validity, bias assessment, phototype-inclusive performance, and clinical relevance within a defined context of use (12, 13, 84, 85, 89–91).

Clinical interpretation and longitudinal monitoring constitute the integrative axis of the framework rather than an additional diagnostic layer. This axis connects systemic biological context, skin-specific molecular biology, functional tissue behavior, imaging-derived structure, and digital phenotype with medical history, anatomical assessment, skin type, environmental exposure, prior procedures, procedural risk, patient goals, and observed treatment response. The clinical value of the model emerges from this integration, not from any isolated measurement.

A central principle of the framework is that the same measurement may carry different evidentiary requirements depending on its context of use. A tool used for documentation requires reproducibility and standardized acquisition. A biomarker used for biological stratification requires evidence of clinical validity. A measurement proposed for treatment selection, risk prediction, outcome monitoring, or claims of rejuvenation requires stronger evidence of clinical utility. This context-dependent interpretation aligns with biomarker frameworks that distinguish analytical validity, clinical validity, and clinical utility, and is especially important in esthetic medicine, where commercial scoring systems and biological-age claims may be adopted before sufficient validation (7–11).

The framework also reduces the risk of overinterpreting isolated measurements. Systemic biological age does not equal skin age (6, 15–18, 24, 26, 27). Skin autofluorescence does not capture all advanced glycation end product species (55, 56). A genetic risk variant does not determine an individual's aging trajectory (60, 61). A wrinkle score does not measure collagen biology (36–38, 86, 87). A digital skin-age estimate does not prove biological rejuvenation (86–88). A single elasticity or transepidermal water loss value does not define treatment suitability (62–65). Interpretive confidence increases when findings from independent diagnostic domains converge toward the same biological or clinical explanation.

At present, this framework should be viewed as a research-oriented and clinically interpretive model, not as a validated decision system. Its immediate value lies in structuring biomarker selection, endpoint development, study design, longitudinal monitoring, and mechanistic interpretation in precision esthetic dermatology. Its future clinical value will depend on prospective studies correlating multilayer biomarker profiles with treatment response, recovery kinetics, complication risk, durability of improvement, patient-reported outcomes, and objective tissue-health endpoints. Responsible translation will require standardized methodology, transparent reporting, phototype-inclusive validation, and avoidance of unvalidated claims of biological rejuvenation (7–14).

**Figure 2:**
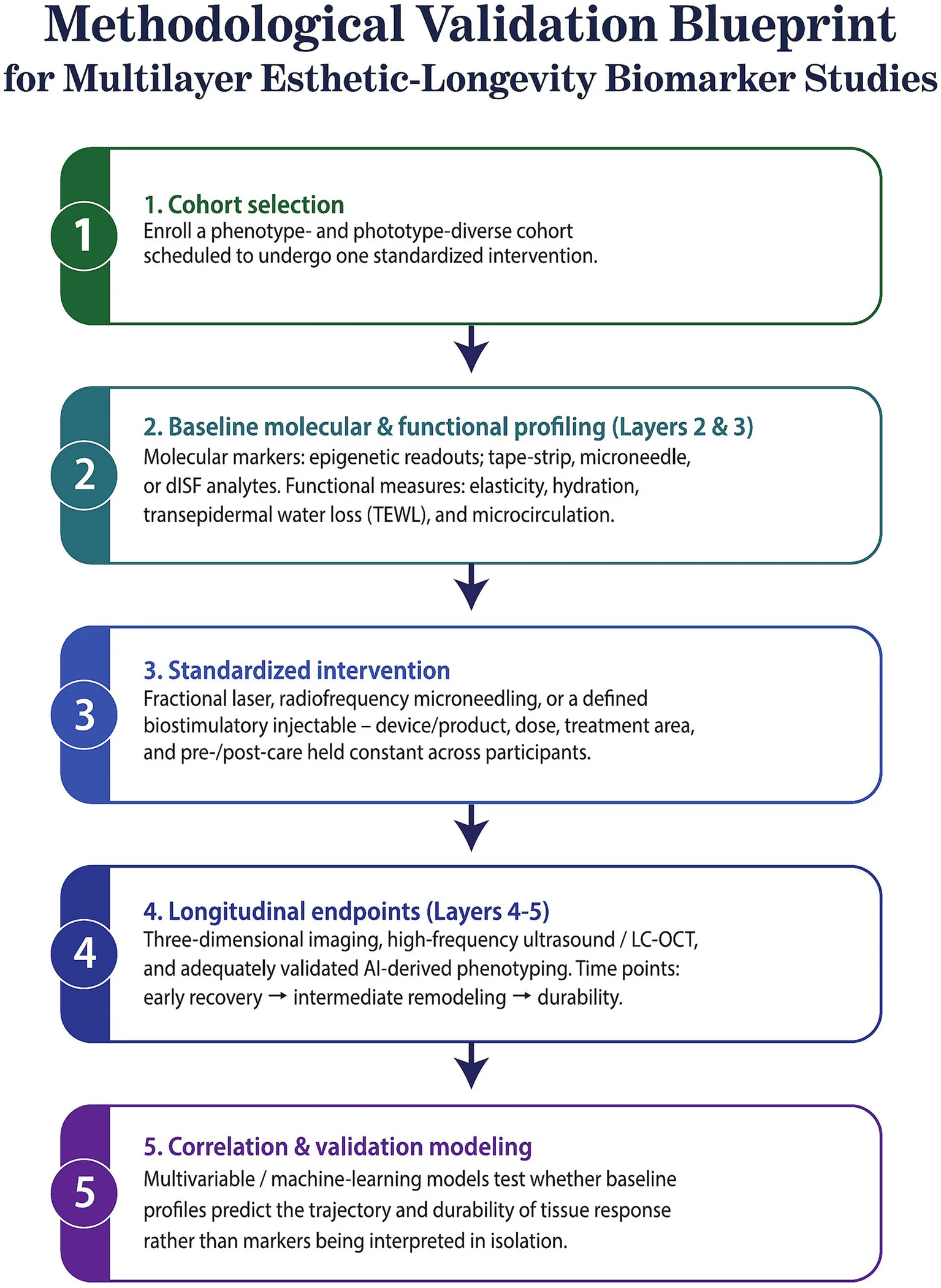
Methodological validation blueprint for future multilayer esthetic-longevity biomarker studies. The blueprint defines a research roadmap for prospectively testing whether baseline multilayer biomarker profiles predict the trajectory and durability of tissue response, and for establishing the analytical validity, clinical validity, and clinical utility of candidate biomarkers within a defined esthetic context of use; it is intended to guide future validation research rather than to direct clinical decisions.

To translate this framework into testable research, we propose a methodological road map for future multilayer esthetic-longevity biomarker studies (Figure 2). It links the five diagnostic layers to a concrete study design—cohort selection, baseline molecular and functional profiling, standardized intervention, longitudinal imaging- and AI-based endpoints, and correlation modeling—and is intended to guide, not prescribe, future work.

## 9 Discussion

Esthetic medicine is increasingly converging with longevity science, regenerative medicine, dermatology, and precision health. Within this convergence, esthetic aging can be understood not merely as a change in appearance, but as an external phenotype of biological aging expressed through skin, soft-tissue, vascular, structural, and regenerative alterations. This shift reframes esthetic assessment from a primarily descriptive evaluation of surface features toward a biologically contextualized interpretation of tissue quality, resilience, and response to intervention (5).

As the field moves toward precision esthetic medicine, diagnostics must extend beyond traditional visual assessment, photography, and expert judgment alone. These tools remain essential, but they primarily characterize phenotype rather than the biological and functional determinants that shape tissue behavior. A precision-oriented approach therefore requires biomarkers and diagnostic modalities capable of linking external phenotype with systemic biological context, local tissue biology, functional capacity, imaging-derived structural information, and longitudinal treatment response. In this setting, biomarkers are not intended to replace clinical expertise, but to support more reproducible, mechanism-informed, and outcome-oriented decision-making (7, 8).

This article provides a critical review of the emerging literature on diagnostics and biomarkers relevant to esthetic longevity medicine and proposes an integrative interpretive framework for their clinical and translational use. The aim is not to define a fixed biomarker panel or a deterministic algorithm, but to organize the available evidence into a clinically meaningful structure. Such a framework may help distinguish between markers that are currently useful for documentation or monitoring, markers that remain primarily investigational, and markers that may eventually support risk stratification, treatment planning, or assessment of biological response once adequately validated (7–11).

A central strength of this approach is that it acknowledges the inherent biological heterogeneity of esthetic aging. Chronological age alone does not explain the marked variability observed in skin quality, inflammatory reactivity, pigmentation risk, extracellular matrix integrity, vascular support, wound-healing dynamics, or durability of treatment response. Patients with similar esthetic phenotypes may differ substantially in systemic inflammatory burden, metabolic health, glycation exposure, oxidative stress, barrier function, or regenerative reserve. Conversely, similar biological risk profiles may manifest differently depending on anatomy, phototype, environmental exposure, prior procedures, and local tissue history. This heterogeneity supports the need for multidomain assessment rather than reliance on any single biomarker or device-derived score (1–5, 36–38, 47, 51, 52, 55–58).

At the same time, the field must recognize important limitations. Many biomarkers discussed in esthetic longevity are supported by biological plausibility rather than direct esthetic-outcome validation. Epigenetic clocks and other biological-age models have transformed longevity research, but most were developed to predict systemic morbidity, mortality, or functional decline rather than local cutaneous aging or procedural response. Similarly, inflammatory, oxidative, glycation-related, senescence-associated, and extracellular matrix markers are mechanistically relevant to skin aging, yet their translation into routine esthetic decision-making remains incomplete. Their use should therefore be considered interpretive and hypothesis-generating unless supported by prospective validation in esthetic populations (7–11, 14).

Imaging, functional testing, and AI-derived digital phenotyping offer substantial promise, but they also illustrate the difference between quantification and validation. A measurement may be reproducible without being clinically actionable; a device-generated score may support documentation without qualifying as a biomarker of biological aging; and an AI-derived skin-age or facial-age estimate may improve phenotypic assessment without proving tissue rejuvenation. The precision of numerical output should not be confused with the validity of biological interpretation. This distinction is especially important in esthetic medicine, where commercial platforms increasingly generate quantitative scores that may influence patient perception, marketing claims, and treatment expectations (7, 8, 86, 87, 89).

Another major limitation is the lack of standardized acquisition protocols and clinically meaningful thresholds. Photography, multispectral imaging, three-dimensional imaging, ultrasound, elasticity testing, barrier measurements, and AI-based tools are all sensitive to technical and biological sources of variation. Lighting, facial expression, device calibration, anatomical site, operator technique, probe pressure, hydration, edema, inflammation, skin phototype, cosmetic use, and timing after treatment can all influence results. Without standardized protocols and test–retest reliability, longitudinal change may reflect measurement variability rather than true tissue modification (9–11, 62–76).

Population diversity remains a critical requirement for future validation. Skin aging, pigmentation, erythema, vascular visibility, scar formation, barrier function, apparent age, and response to energy-based procedures differ across phototypes, ethnic backgrounds, sexes, ages, and environmental exposure patterns. Biomarkers or algorithms developed in narrow populations may not generalize across diverse esthetic populations. This is particularly relevant for AI-derived outputs, pigmentation analysis, erythema detection, vascular assessment, and apparent-age estimation. Phototype-inclusive validation should therefore be considered a foundational scientific requirement for esthetic longevity diagnostics (9–11, 14, 89, 91).

AI is likely to play an increasingly important role in esthetic longevity diagnostics, but its role should be carefully defined. AI can support standardized phenotyping, longitudinal tracking, image analysis, multimodal integration, and pattern recognition across complex datasets. However, AI does not solve the problem of validation. An AI-derived output is only as meaningful as the data, ground truth, acquisition protocol, population diversity, and clinical endpoint on which it is built. In esthetic medicine, where many outputs are continuous, subjective, and phenotype-based, AI should currently be regarded as a tool for enhancing measurement and interpretation, not as proof of biological rejuvenation or as an autonomous treatment-selection system (12, 13, 84, 85, 89, 91).

The principal future direction should therefore be the development of prospectively validated, multimodal, outcome-linked research. Studies should define the intended context of use before testing a biomarker, standardize acquisition protocols, include diverse populations, assess reproducibility and sensitivity to change, and correlate biomarker findings with clinically meaningful outcomes. These outcomes should include not only visible improvement, but also recovery kinetics, complication risk, durability, tissue quality, patient-reported outcomes, and, where appropriate, mechanistic indicators of remodeling or repair. Only through such studies can biomarkers move from conceptual relevance to clinical utility (9–14). Beyond episodic assessment, the emergence of wearable and continuous digital biomarkers — including sensors capable of longitudinally tracking barrier function, hydration, and microenvironmental parameters — may eventually enable dynamic, real-world monitoring of skin aging and treatment response rather than reliance on isolated time-point measurements (20, 49).

A scientifically sound future perspective should also preserve appropriate restraint in terminology. Changes in wrinkle depth, pigmentation, dermal thickness, ultrasound echogenicity, elasticity, transepidermal water loss, inflammatory markers, methylation age, or digital apparent age may be clinically relevant, but they should not automatically be described as biological rejuvenation. Claims of rejuvenation, age reversal, or regenerative optimization require evidence that the measured change reflects a validated biological aging process and is linked to durable clinical benefit. Until such evidence is available, biomarkers should be used to support interpretation, stratification, monitoring, and research rather than deterministic clinical claims (7–11).

In practical terms, the near-term role of esthetic longevity diagnostics is to improve the quality of clinical reasoning and research methodology. Biomarkers can help characterize baseline tissue status, identify potentially modifiable biological risk factors, document longitudinal response, and generate more objective outcome data. In the intermediate term, validated biomarker profiles may support patient stratification, procedural timing, combination-treatment planning, and monitoring of treatment durability (7–11). In the long term, integrated diagnostic models may enable a more adaptive form of esthetic medicine in which interventions are selected, sequenced, and maintained according to tissue biology, functional reserve, and longitudinal response rather than external phenotype alone (7–14).

## 10 Conclusion

Overall, diagnostics and biomarkers offer an opportunity to strengthen the scientific foundation of esthetic medicine. Their value lies not in replacing clinical artistry, anatomical expertise, or esthetic judgment, but in providing a more rigorous biological and translational framework for interpreting external phenotype. If developed with appropriate validation, standardization, transparency, and clinical restraint, esthetic longevity diagnostics may help define a more precise and scientifically credible discipline at the interface of aging biology, regenerative medicine, dermatology, and esthetic practice. Future research should evaluate whether multilayer diagnostic profiling can improve treatment selection, procedural sequencing, recovery, and durability of esthetic outcomes, moving precision esthetic longevity from a conceptual framework toward clinical reality.
